# Changes overtime in primary and secondary empty sella: a comparative real-world study

**DOI:** 10.1007/s40618-026-02931-2

**Published:** 2026-06-08

**Authors:** Nicolò Bacchi, Francesca Paglia, Vanessa Caccin, Bruno Madeo, Gianluca Margiotta, Maria Chiara Decaroli, Maria Laura Monzani, Chiara Diazzi, Sara De Vincentis, Vincenzo Rochira

**Affiliations:** 1 Endocrinology, Department of Biomedical, Metabolic and Neural Sciences, University of Modena and Reggio Emilia, Modena, Italy; 2 Unit of Endocrinology, Department of Medical Specialties, Azienda Ospedaliero-Universitaria di Modena, Ospedale Civile di Baggiovara, Modena, Italy

**Keywords:** Pituitary, Central hypogonadism, Hypopituitarism, Empty sella syndrome, GH deficiency, Central adrenal insufficiency

## Abstract

**Purpose:**

Primary (PES) and Secondary (SES) Empty Sella (ES) show similar radiological appearances, but they may be associated with variable degrees of pituitary dysfunction. Long-term comparative data are limited. This study aimed to compare baseline and longitudinal endocrine outcomes of PES and SES in a large single-center cohort.

**Methods:**

We conducted a retrospective, longitudinal study including adults with ES followed at a tertiary endocrinology center between 2007 and 2025. ES was classified as PES or SES based on clinical history. Clinical and neuroradiological features, comorbidities, and pituitary hormonal assessments were collected at baseline and during follow-up.

**Results:**

A total of 258 participants were included (64.3% women; median age 51.5 years; median follow-up 58.5 months). PES was more prevalent than SES (61.6% vs. 38.4%). At baseline, 41.1% of patients had at least one pituitary hormone deficiency, significantly more frequent in SES than PES (62.6% vs. 27.7%), with gonadotropic deficiency the most common alteration. During follow-up, pituitary function remained stable in most PES patients (83.3%), with a very low incidence of new deficiencies. In contrast, SES showed a significantly higher risk of developing new corticotropic, thyrotropic, and gonadotropic deficiencies. Arginine vasopressin deficiency occurred exclusively in SES.

**Conclusion:**

In this large longitudinal cohort, PES and SES showed distinct clinical and endocrine evolution. While PES was generally stable course over time, SES was associated with a higher risk of progressive pituitary dysfunction. These findings support systematic baseline endocrine evaluation in all ES patients and a tailored follow-up strategy, with closer surveillance for SES.

**Supplementary Information:**

The online version contains supplementary material available at 10.1007/s40618-026-02931-2.

## Introduction

Empty sella (ES) is a radiological condition characterized by herniation of the subarachnoid space into the sella turcica, leading to pituitary flattening and partial or complete filling of the sella with cerebrospinal fluid (CSF) [1, 2]. ES is classified as primary (PES), occurring without prior pituitary pathology, or secondary (SES), resulting from pituitary damage due to surgery, radiotherapy, apoplexy, hypophysitis, trauma, or other insults [1–4]. Radiologically, ES may be partial or complete depending on whether less or more than 50% of the sellar cavity is filled with CSF, with pituitary thickness ≤ 2 mm in the latter case [5, 6].

Although PES and SES often share similar imaging features, they differ substantially in underlying pathophysiology and clinical context [2, 3, 7]. PES is generally considered a multifactorial condition, associated with sellar diaphragm incompetence, chronic intracranial hypertension, and dynamic pituitary volume changes [1, 5, 7, 8], as well as comorbidities such as obesity and hypertension [1, 9, 10]. PES is often detected incidentally, whereas SES typically occurs in patients with known or suspected pituitary disease and reflects structural damage to the gland [1, 2].

Both forms may be complicated by hypopituitarism, which is usually more frequent and severe in SES [2–4, 7]. However, the natural history of ES remains incompletely defined. Longitudinal studies comparing PES and SES are limited, and available studies have reported heterogeneous results regarding the stability or progression of pituitary dysfunction, particularly in PES [3, 6, 10. Given these uncertainties, current recommendations on endocrine evaluation and follow-up are largely based on expert opinion rather than robust longitudinal evidence and no evidence-based guidelines are currently available [2, 7, 8].

Given the overlap in radiological appearance but differences in pathophysiology between PES and SES, a systematic comparison of their clinical, radiological, and endocrine features over time is needed [11]. Besides, long-term data directly comparing the natural history of PES and SES are still limited and often conflicting. In this context, the present study was designed to provide a comprehensive baseline and longitudinal assessment of a large single-center cohort of patients with ES, with the aim of better characterizing the clinical and endocrine course of PES and SES in real-world practice. By providing long-term comparative data on these two conditions, this study seeks to improve understanding of ES natural history and optimizing patient management.

## Materials and methods

### Study design

We conducted a retrospective, longitudinal study belonging to the Research Project ‘Pituitary disorders in the Modena population: registry of clinical and epidemiological data of patients attending the Hypothalamic-Pituitary Disorders Outpatient Clinic’ aiming to investigate real-life data in the single center of the Unit of Endocrinology (EndoMO), University of Modena and Reggio Emilia, Azienda Ospedaliero–Universitaria of Modena, Italy. All clinical data from patients with hypothalamo-pituitary disorders are systematically and prospectively recorded in the DataPit, an institutional database established within this research project and specifically designed for the standardized collection and longitudinal analysis of data from all patients with pituitary diseases. All patients consecutively evaluated between January 2007 and March 2025 with a radiological diagnosis of ES were considered for inclusion in the study. According to neuroradiological findings patients included in the study were subdivided into two groups: PES (Group 1) and SES (Group 2).

Data were collected at two time points: V0, defined as the first access to our service (often coinciding with the radiological diagnosis of ES), and V1, the last available endocrinological follow-up. At both visits, demographic information, medical history, clinical features, anthropometric measurements, radiological characteristics, and endocrine function were assessed. The duration of the follow-up was expressed in months between V0 and V1.

### Participants

All patients with ES and followed at EndoMO were considered eligible to enter the study according to the following inclusion and exclusion criteria.

Inclusion criteria were age ≥ 18 years, radiological evidence of ES on computed tomography (CT) and/or magnetic resonance imaging (MRI), and a follow-up duration of at least 2 months.

Exclusion criteria were incomplete clinical or radiological records, uncertain classification between PES and SES.

### Clinical evaluation

Data from clinical evaluations performed at each visit were extracted from medical charts and entered into the large DataPit database including; age at diagnosis, sex, reason for performing MRI/CT imaging, symptoms, past medical history (e.g. history of traumatic brain injury), number of pregnancies (for females), comorbidities, medications and results of radiological and biochemical examinations.

*Signs and symptoms*: asthenia, fatigue, menstrual cycle disorders, low libido, erectile dysfunction, headache, rhinorrhea, visual deficit, intracranial hypertension/papilledema, neurological disorders, polyuria, polydipsia.

*Physical examination*: weight, height, body mass index (BMI).

### Neuroradiological evaluation

All patients underwent contrast-enhanced MRI and/or CT scanning of the sella turcica to confirm the diagnosis of ES and to distinguish between complete and partial ES. MRI was performed at a field strength of at least 1.5 Tesla (Siemens MAGNETOM Aera 1.5 Tesla), and both T1-weighted and T2-weighted images were acquired. CT examinations were performed using a Philips Incisive CT scanner (vMRC tube, 128-slice acquisition, 72-cm bore, 40-mm coverage, 0.35-s rotation time, generator power 105 kW, 70–140 kV), with intravenous contrast administration when indicated.

Less than 2% of patients diagnosed with ES at neuroradiological services outside our hospital were included only after internal revision of the imaging study or repetition of imaging at the Neuroradiological Unit of Azienda Ospedaliero-Universitaria of Modena.

ES was diagnosed on sella turcica or brain CT and/or MRI according to established criteria: partial ES was defined as < 50% of the sella turcica filled with CSF and pituitary thickness > 2 mm, while complete ES was defined as > 50% CSF filling with pituitary thickness ≤ 2 mm (2–4).

The distinction between PES and SES was based on the exclusion of any previous pituitary or sellar disease for PES, whereas SES was diagnosed in presence of pituitary injury due to surgery, radiotherapy, apoplexy, Sheehan’s syndrome, hypophysitis, trauma, infectious, granulomatous disease or any other disease of the hypothalamic-pituitary region damaging the pituitary [1, 2, 4].

### Hormonal parameters

All hormonal assessments were performed in the same hospital laboratory, in the morning after an overnight fast, using commercially available immunometric assays (see Supplemental materials). For each patient, anterior and posterior pituitary function was evaluated both at V0 and V1 including: adrenocorticotropic hormone (ACTH), serum and 24-hour urinary cortisol, thyroid-stimulating hormone (TSH), free thyroxine (fT4), free triiodothyronine (fT3), follicle-stimulating hormone (FSH), luteinizing hormone (LH), estradiol, testosterone (only in men), progesterone (only in women), insulin-like growth factor 1 (IGF-1), prolactin, serum sodium, blood urea nitrogen, and glucose.

The presence of hormonal deficiencies (i.e. central adrenal insufficiency [AI], central hypothyroidism [CH], central hypogonadism, growth hormone [GH] deficiency, Arginine Vasopressin Deficiency [AVD] or central diabetes insipidus, prolactin alterations) was detected according to standardized biochemical and dynamic criteria (see Supplemental material).

For each patient, the number of impaired hormonal axes (0–6) was recorded. Endocrine function was reassessed at V1 to evaluate stability, the new onset of pituitary deficiencies, or recovery.

### Comorbidities

Major comorbidities were systematically assessed for each patient. Specifically, the presence of metabolic syndrome, arterial hypertension, diabetes mellitus, and major adverse cardiovascular events (MACE) was recorded according to standard clinical criteria.

### Statistical analysis

Data are presented as median and Interquartile Range (IQR) for continuous variables and as number and percentage for categorical variables.

The non-parametric Mann–Whitney U test was used for comparisons of continuous variables since they resulted not normally distributed at the Kolmogorov–Smirnov test. Categorical variables were compared by Pearson’s Chi-squared test.

Linear regression was used to examine the association between continuous variables; significant results were expressed through the R^2^ coefficient. Bivariate ordinal logistic regression was used to explore contributing factors for more than 2 hormone pituitary deficiencies; thereafter, multivariate regression analysis using a backward elimination method was applied to the data with *p* < 0.1 as the criterion for a variable to enter the model. Results of proportional odd models were expressed through odds ratio (OR) and 95% confidence interval.

Statistical analyses were performed using the Statistical Package for the Social Sciences’ (SPSS) software for Windows (version 29.0; SPSS Inc, Chicago, IL). For all comparisons, *p* < 0.05 was considered statistically significant.

## Results

**Fig. 1:**
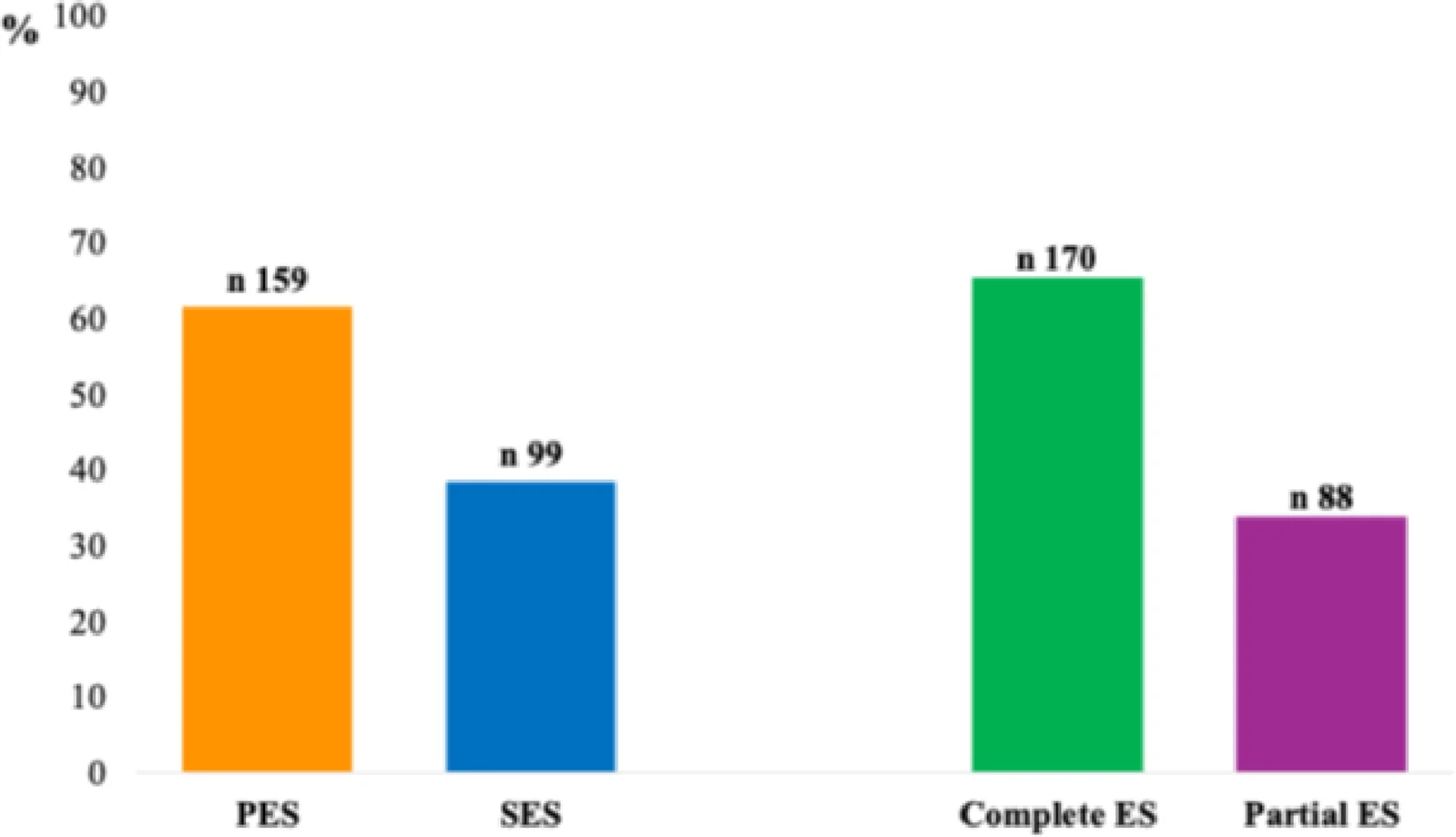
Distribution of patients according to Empty Sella type and grade (n; %). Primary Empty Sella (PES), Secondary Empty Sella (SES)

A total of 258 patients with ES were included in the study. The demographic and clinical characteristics of the entire cohort are summarized in Table 1. The median age at diagnosis was 51.5 years, the median follow-up period was 58.5 months (about 5 years); women represented almost two-thirds of the cohort (64.3%) (Table 1). PES was more prevalent (n 159; 61.6%) than SES (n 99; 38.4%) (Table 1; Fig. 1). Overall, 54.7% of patients were referred for the suspicion of pituitary disease, whereas 45.3% were diagnosed incidentally (Table 1). Complete ES (66%) was more frequently diagnosed than partial ES (34%) at radiological examination (Table 1; Fig. 1).

**Table 1:** Descriptive characteristics of the entire cohort of patients with Empty Sella (ES). Continuous variables are reported as median values with interquartile ranges in parenthesis (IQR), and categorical variables as absolute frequencies (n) and percentages (%) in parenthesis

	Patients (*n* 258)
Demographic characteristics	
Age (years)	51.5 (40.7–62.1)
Sex n, (%) Male Female	92 (35.7%) 166 (64.3%)
Anthropometric characteristics	
Weight (kg)*	76.00 (62.3–86.0)
BMI (kg/m^2^)**	26.77 (23.7–31.6)
Clinical characteristics	
PES n, (%)	159 (61.6%)
SES n, (%)	99 (38.4%)
Partial ES n, (%)	88 (34%)
Complete ES n, (%)	170 (66%)
Diagnosis characteristics and clinical conditions at the time of diagnosis	
Incidental findings n, (%)	117 (45.3%)
Clinical suspicion of pituitary disease n, (%)	141 (54.7%)
Symptoms	
Headache n, (%)	61 (23.6%)
Asthenia n, (%)	33 (12.8%)
Polyuria and polydipsia n, (%)	15 (5.8%)
Traumatic brain injury n, (%)	17 (6.6%)
Visual field defect n, (%)	21 (8.1%)
Intracranial hypertension n, (%)	10 (3.9%)
Pituitary dysfuncion	
Absence of pituitary hormone deficiencies	152 (58.9%)
Presence of pituitary hormone deficiencies	106 (41,1%)
Duration of follow-up (months)***	58.5 (36.1–90.0)

Body mass index (BMI), Primary Empty Sella (PES), Secondary Empty Sella (SES), Growth Hormone (GH), Adrenocorticotropic Hormone (ACTH), Thyroid-stimulating Hormone (TSH), Follicle-stimulating Hormone (FSH), Luteinizing Hormone (LH), Prolactin (PRL). *Weight was available for 185 out of 258 patients; **BMI was available for 169 out of 258 patients; *** Duration of follow up was available for 227 out of 258 patients

At diagnosis of ES, headache was the most common symptom occurring in 23.6% of patients followed by asthenia (12.8%), visual field defects (8.1%), and mild to severe polydipsia (5.8%) (Table 1). Besides, a history of traumatic brain injury and intracranial hypertension was recorded in 6.6% and 3.9% of cases, respectively (Table 1).

**Fig. 2:**
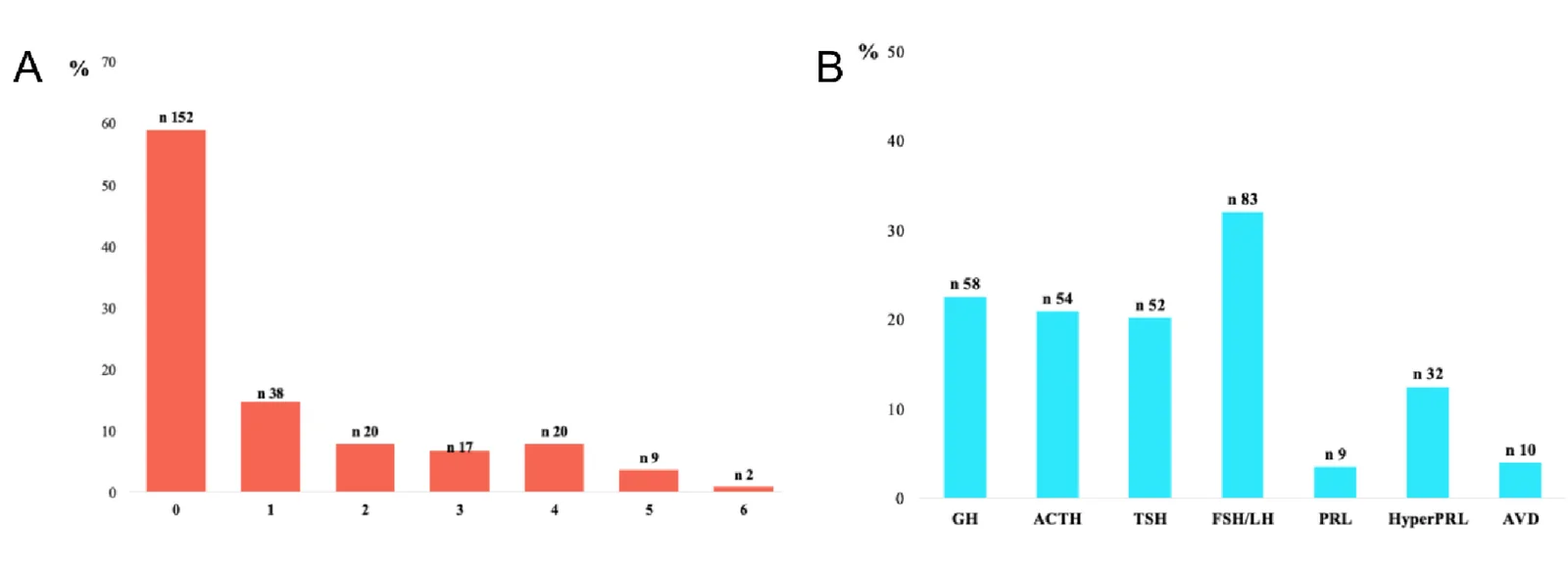
Prevalence of pituitary hormone deficiencies among patients with Empty Sella (ES) in terms of total number of pituitary deficiencies (n; %) (Fig. 2A) and in term of pituitary axes involvement (Fig. 2B). Growth hormone (GH), adrenocorticotropic hormone (ACTH), thyroid-stimulating hormone (TSH), follicle-stimulating hormone (FSH), luteinizing hormone (LH), prolactin (PRL). hyperprolactinemia (HyperPRL), Arginine Vasopressin Deficiency Diabetes insipidus (AVD)

Endocrine evaluation revealed that 41.1% of patients presented with at least one pituitary hormone deficiency (Table 1; Fig. 2A). A single-axis impairment was observed in 14.7%, while multiple deficiencies were less common (Fig. 2A). The gonadotropic axis was the most frequently affected, with FSH/LH deficiency documented in 32% of cases followed by GH (22.5%; 29/56 patients were diagnosed based on the presence of > 3 pituitary hormone deficiencies, 27/56 patients underwent dynamic stimulation testing), ACTH (20.0%), and TSH (20.2%) deficiency, being AVD and hypoprolactinaemia less frequent (< 4%) (Fig. 2B). Of note, complete anterior hypopituitarism was rare (n 2; 0.8%) and occurred only in patients with SES. Finally, hyperprolactinaemia was found in 12.4% of patients with ES (Fig. 2B).

No significant differences were found between partial and complete ES for all the variables considered.

At ordinal logistic regression analysis male sex, clinical signs and/or symptoms prompting the diagnosis, having SES, and an older age both at the time of diagnosis and of first visit were identified as significant predictors for the development of more than 2 hormone pituitary deficiencies, while BMI, the type of ES (Complete/Partial), and the total number of comorbidities were not included in the model (Table 2).

**Table 2:** Determinants of more than two hormone pituitary deficiencies in patients with ES at the time of diagnosis. Results of proportional odd models were expressed through odds ratio (OR) and 95% confidence interval

Variable	Dependent variable: more than 2 hormone pituitary deficiencies	
	OR [95%CI]	*P* value
Age at diagnosis	0.92 [0.86–0.98]	0.01
Age at first visit (v0)	1.09 [1.02–1.18]	0.02
Sex	4.48 [1.71–11.73]	0.00
BMI	1.05 [0.97–1.13]	0.23
Type of ES (PES/SES)	0.22 [0.07–0.65]	0.01
Grade of ES (Complete/Partial)	1.81 [0.68–4.84]	0.24
Clinical Diagnosis	0.23 [0.06–0.86]	0.03
Total number of comorbidities	0.87 [0.62–1.23]	0.42

Body mass index (BMI), Empty Sella (ES), Primary Empty Sella (PES), Secondary Empty Sella (SES)

### Sex differences

Of the 258 patients with ES, 92 were males and 166 females (Table 3). Women were younger at diagnosis (*p* = 0.025) and more frequently had primary ES (*p* = 0.004) than men (Table 3). Incidental diagnosis was more common in females, whereas clinical suspicion of pituitary disease predominated in males (*p* = 0.002) (Table 3).

**Table 3:** Descriptive characteristics of patients with Empty Sella (ES) according to sex. Continuous variables are reported as median values with interquartile ranges in parenthesis (IQR), and categorical variables as absolute frequencies (n) and percentages (%) in parenthesis

	Males (*n* 92)	Females (*n* 166)	*p*-value
Demographic characteristics			
Age (years)	51.9 (37.3–62.8)	47.6 (40.4–59.5)	0.44
Anthropometric characteristics			
BMI (kg/m^2^)*	26.6 (24.8–30.6)	26.8 (23.0–32.7)	0.70
ES classification			
Primary ES (PES)	46 (50%)	113 (68.1%)	0.00
Secondary ES (SES)	46 (50%)	53 (31.9%)	
Partial ES n, (%)	36 (39%)	51 (30.7%)	0.13
Complete ES n, (%)	54 (58.6%)	115 (69.3%)	
Diagnosis characteristics and clinical conditions at the time of diagnosis			
Incidental findings n, (%)	30 (32.6%)	87 (52.4%)	0.00
Clinical suspicion of pituitary disease	62 (67.4%)	79 (47.6%)	
Symptoms			
Headache n, (%)	16 (17.4%)	45 (27.1%)	0.08
Traumatic brain injury n, (%)	9 (9.8%)	8 (4.8%)	0.12
Visual field defect n, (%)	10 (10.9%)	11 (6.6%)	0.23
Intracranial hypertension n, (%)	1 (1.1%)	9 (5.4%)	0.08
Asthenia n, (%)	11 (12.0%)	22 (13.3%)	0.76
Polyuria and polydipsia n, (%)	7 (7.6%)	8 (4.8%)	0.36
Pituitary dysfunction			
Absence of pituitary hormone deficiencies	39 (42.4%)	115 (69.3%)	< 0.001
Presence of pituitary hormone deficiencies	53 (57.6%)	51 (30.7%)	
GH deficiency n, (%)	31 (33.7%)	26 (15.7%)	< 0.001
ACTH deficiency n, (%)	29 (31.5%)	25 (15.1%)	0.00
TSH deficiency n, (%)	26 (28.3%)	26 (15.7%)	0.02
FSH/LH deficiency n, (%)	45 (48.9%)	38 (22.9%)	< 0.001
Hyperprolactynemia	14 (15.2%)	18 (11.0%)	0.31
AVD	6 (6.5%)	4 (2.4%)	0.10
Duration of follow-up (months)**	66.1 (42.9–101.1)	53.5 (32.3–88.9)	0.02

Body mass index (BMI), Primary Empty Sella (PES), Secondary Empty Sella (SES), Growth Hormone (GH), Adrenocorticotropic Hormone (ACTH), +Tthyroid-stimulating Hormone (TSH), Follicle-stimulating Hormone (FSH), Luteinizing Hormone (LH), Arginine Vasopressin Deficiency (AVD). *BMI was available for 169 out of 258 patients; ** Duration of follow-up was available for 227 of 258 patients: 85 were males and 142 were females

Absence of pituitary hormone deficiencies was significantly higher in females (*p* < 0.001) than in males, while multiple hormonal deficits, particularly corticotropic, thyrotropic, and gonadotropic, were more prevalent in males (*p* ≤ 0.001) (Table 3), as further substantiated by the results of logistic regression (Table 2).

Other clinical features, including headache and visual disturbances, did not differ by sex (Table 3).

### Differences between PES and SES

**Fig. 3:**
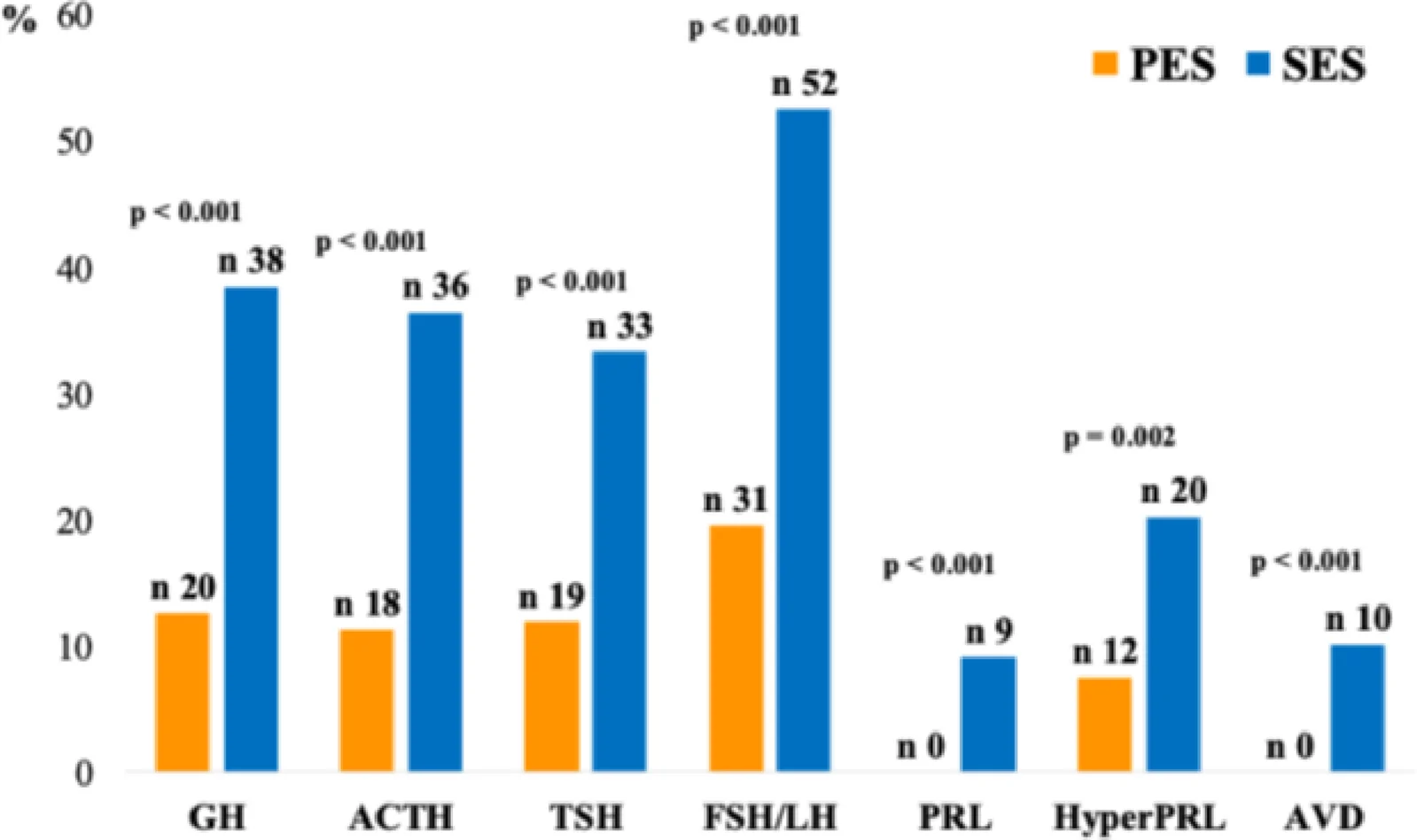
Prevalence of PES and SES according to clinical presentation (clinical suspicion vs. incidental finding) at the time of diagnosis. Primary Empty Sella (PES), Secondary Empty Sella (SES)

Patients with PES were significantly older at diagnosis (*p* = 0.025) and more frequently female (*p* = 0.004) compared with those with SES (Table 4). SES was more often diagnosed in the context of clinical suspicion (87.9%), whereas PES was typically detected incidentally (*p* < 0.001) (Table 4; Fig. 3). Diagnosis of ES was more frequently obtained from MRI in SES than PES (*p* = 0.003); the distribution between complete and partial ES did not differ between PES and SES (*p* = 0.532) (Table 4).

**Table 4:** Comparison of baseline characteristics between patients with PES and SES. Continuous variables are reported as median values with interquartile ranges in parenthesis (IQR), and categorical variables as absolute frequencies (n) and percentages (%) in parenthesis

	PES (*n* 159)	SES (*n* 99)	*p*-value
Demographic characteristics			
Age (years)	52.86 (42.1–64.4)	46.13 (36.0–60.0)	0.03
Sex n, (%) Male Female	46 (28.9%) 113 (71.1%)	46 (46.5%) 53 (53.5%)	0.00
Anthropometric characteristics			
Weight (kg)*	75.00 (61.5–85.5)	77 (63.3–88.3)	0.37
BMI (kg/m^2^)**	27.01 (24.2–31.6)	26.28 (23.4–31.5)	0.43
Neuroradiological characteristics			
Partial ES n, (%)	56 (35.4%)	31 (31.3%)	0.53
Complete ES n, (%)	103 (64.6%)	68 (67.7%)	
Diagnosis characteristics and clinical conditions at the time of diagnosis			
Incidental findings n, (%)	105 (66%)	12 (12.1%)	< 0.001
Clinical suspicion of pituitary disease	54 (34%)	87 (87.9%)	
Radiological procedure			
CT n, (%)	34 (21%)	7 (7%)	0.00
MRI n, (%)	125 (79%)	92 (93%)	
Symptoms			
Headache n, (%)	42 (26.4%)	19 (19.2%)	0.18
Traumatic brain injury n, (%)	7 (4.4%)	10 (10.1%)	0.07
Visual field defect n, (%)	8 (5%)	13 (13.1%)	0.02
Intracranial hypertension n, (%)	9 (5.7%)	1 (1%)	0.06
Asthenia n, (%)	17 (10.7%)	16 (16.2%)	0.20
Polyuria and polydipsia n, (%)	6 (3.8%)	9 (9.1%)	0.08
Pituitary dysfunction			
Absence of pituitary hormone deficiencies	115 (72.3%)	37 (37.4%)	< 0.001
Presence of pituitary hormone deficiencies	42 (27.7%)	62 (62.6%)	
Reproductive data in women			
Pregnancies n, (%)*** 0 ≥ 1	21 (33.9%) [41 (66.1%)]	11 (30.6%) [25 (69.4%)]	0.32
Eumenorrheic n, (%)	34 (30.1%)	12 (22.6%)	0.16
Oligomenorrhoea n, (%)	17 (15%)	14 (26.4%)	
Menopause n, (%)	62 (54.9%)	27 (50.9%)	
Prevalence of comorbidities			
Metabolic syndrome n, (%)	30 (18.9%)	18 (18.2%)	0.89
Hypertension n, (%)	77 (48.4%)	29 (29.3%)	0.00
MACE n, (%)	19 (11.9%)	4 (4%)	0.03
Diabetes mellitus n, (%)	17 (10.7%)	9 (9.1%)	0.68
Duration of follow-up (months)****	52.0 (31.2–75.1)	70.9 (47.4–107.3)	0.00

Primary Empty Sella (PES), Secondary Empty Sella (SES), Body Mass Index (BMI), Computed Tomography (CT), Magnetic Resonance Imaging (MRI), Empty Sella (ES), Growth Hormone (GH), Adrenocorticotropic Hormone (ACTH), Thyroid-stimulating Hormone (TSH), Follicle-stimulating Hormone (FSH), Luteinising Hormone (LH), Prolactin (PRL), Major Adverse Cardiovascular Events (MACE). *Weight was available for 101 out of 159 patients with PES and 84 out of 99 patients with SES; **BMI was available for 89 out of 159 patients with PES and 80 out of 99 patients with SES; ***Pregnancy was available for 62 out of 113 female patients with PES and 36 out of 53 female patients with SES; **** Duration of follow-up was available for 132 out of 159 with PES and 95 out of 99 patients with SES

**Fig. 4:**
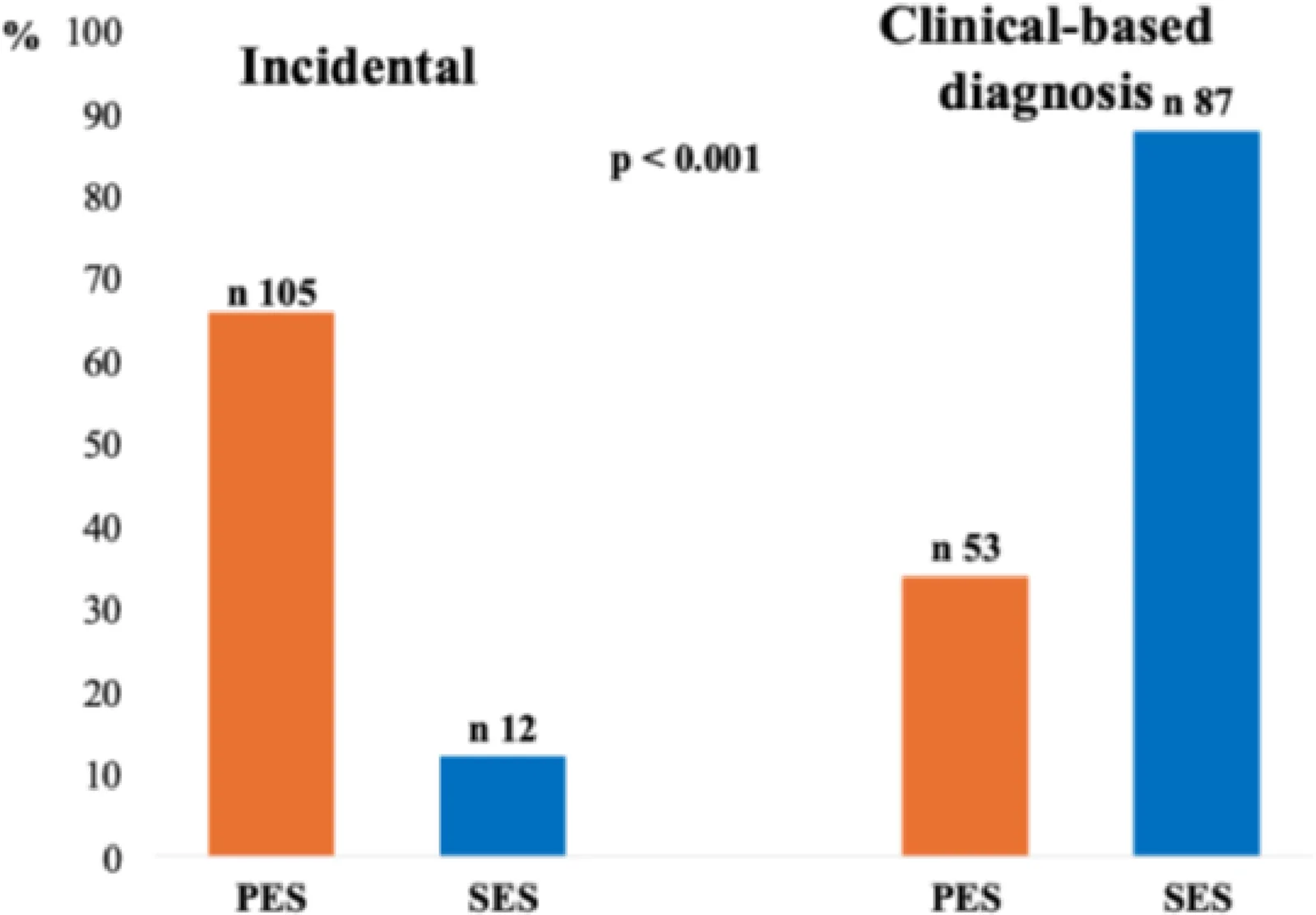
Prevalence of pituitary axes alterations in PES and SES. Primary Empty Sella (PES), Secondary Empty Sella (SES), Growth hormone (GH), adrenocorticotropic hormone (ACTH), thyroid-stimulating hormone (TSH), follicle-stimulating hormone (FSH), luteinizing hormone (LH), prolactin (PRL). hyperprolactinemia (HyperPRL), Arginine Vasopressin Deficiency Diabetes insipidus (AVD)

Clinical manifestations at the time of diagnosis differed between PES and SES since visual field defects were more common in SES (*p* = 0.021) (Table 4). Besides, preservation of pituitary function was observed in 72.3% of PES but only in 37.4% of SES (*p* < 0.001) while multiple pituitary deficiencies were significantly more prevalent in SES, involving GH, ACTH, TSH, and gonadotropins (*p* < 0.001) (Table 4; Fig. 4). AVD occurred exclusively in SES (*p* < 0.001), in line with the higher prevalence of polyuria and polydipsia in this subgroup (Table 4; Fig. 4).

Regarding comorbidities, patients with PES showed a higher prevalence of arterial hypertension (*p* = 0.002) and MACE (*p* = 0.030) (Table 4).

Exploring SES aetiology, post-surgical cases represented the majority (57%), followed by pituitary apoplexy (17%) and post-traumatic forms (10%), other causes being less frequent (Table 5).

**Table 5:** Causes of Secondary Empty Sella (SES)

Cause	*n* (%)
Post-surgical	56 (57%)
Post-radiotherapy	4 (4%)
Post-pharmacological therapy	1 (1%)
Post-traumatic	10 (10%)
Pituitary adenoma apoplexy	17 (17%)
Sheehan’s syndrome	2 (2%)
Lymphocytic hypophysitis	1 (1%)
Infectious causes	1 (1%)
Granulomatous diseases	3 (3%)
Other	4 (4%)

No significant differences were found between PES and SES for BMI and grade of ES (Partial/Complete), as well as for all symptoms at diagnosis with the exception of visual field defects (Table 4).

### Longitudinal clinical and endocrine comparison between PES and SES

The follow-up duration ranged from 36 to 90 months (Table 1), reflecting a notably long observation period. During follow-up, both clinical presentation and pituitary function remained largely stable, although clear differences emerged between PES and SES (Tables 6 and 7).

**Table 6:** Changes of pituitary function and clinical features observed at baseline (V0) and at last follow-up visit (V1) in patients with PES and SES: comparison were made between the two groups for each outcome category (stable, onset, recovery)

Hormonal changes from baseline	PES (*n* 132)			SES (*n* 95)			*p*-value
	Stable	New Onset	Recovery	Stable	New Onset	Recovery	
Total pituitary deficit n, (%)	110 (83.3%)	12 (9.1%)	10 (7.6%)	66 (69.5%)	20 (21.1%)	9 (9.5%)	0.03
Total anterior pituitary deficit n, (%)	110 (83.3%)	12 (9.1%)	10 (7.6%)	68 (71.6%)	20 (21.1%)	7 (7.4%)	0.04
GH deficiency n, (%)	127 (96.2%)	2 (1.5%)	3 (2.3%)	88 (92,6%)	6 (6.3%)	1 (1.1%)	0.13
ACTH deficiency n, (%)	123 (93.2%)	5 (3.8%)	4 (3.0%)	85 (89.4%)	7 (7.4%)	3 (3.2%)	0.49
TSH deficiency n, (%)	127 (96.2%)	4 (3.0%)	1 (0.8%)	83 (87.3%)	11 (11.6%)	1 (1.1%)	0.04
FSH/LH deficiency n, (%)	127 (96.2%)	3 (2.3%)	2 (1.5%)	86 (90.5%)	5 (5.3%)	4 (4.2%)	0.21
AVD n, (%)	132 (100%)	0 (0%)	0 (0%)	92 (96.8%)	1 (1.1%)	2 (2.1%)	0.12
Hyperprolactinemia n, (%)	126 (95.5%)	3 (2.3%)	3 (2.3%)	87 (92%)	3 (3.2%)	5 (5.3%)	0.44
Clinical changes from baseline	Stable	New Onset	Recovery	Stable	New Onset	Recovery	
Neurological symptoms n, (%)	128 (97%)	0 (0%)	4 (3%)	94 (98.9%)	1 (1.1%)	0 (0%)	0.12
Visual field defect n, (%)	129 (97.8%)	0 (0%)	3 (2.3%)	91 (95.8%)	0 (0%)	4 (4.2%)	0.41
Headache n, (%)	115 (87.1)	9 (6.8%)	8 (6%)	77 (81%)	7 (7.4%)	11 (11.6%)	0.32
Asthenia n, (%)	117 (88.6%)	6 (4.6%)	9 (6.8%)	75 (79%)	10 (10.5%)	10 (10.5%)	0.12
Decreased libido n, (%)	129 (97.8%)	1 (0.8%)	2 (1.5%)	85 (89.4%)	3 (3.2%)	7 (7.4%)	0.03

Primary empty Sella (PES), Secondary Empty Sella (SES), growth Hormone (GH), Adrenocorticotropic Hormone (ACTH), Thyroid-stimulating Hormone (TSH), Follicle-stimulating Hormone (FSH), Luteinizing Hormone (LH), Arginine Vasopressin Deficiency (AVD

**Table 7:** Presence/absence of of pituitary hormone deficiencies observed at baseline (V0) and at last follow-up visit (V1) in patients with PES (Part 1) and SES (Part 2).

PES (*n* 132)					*p*-value
Pituitary deficiencies at baseline	No		Yes		
Changes from baseline	Stable	New Onset	Persistance	Recovery	
GH deficiency n, (%)	110 (83.3%)	2 (1.5%)	17 (12.9%)	3 (2.3%)	0.04
ACTH deficiency n, (%)	109 (82.6%)	5 (3.8%)	14 (10.6%)	4 (3.0%)	< 0.001
TSH deficiency n, (%)	109 (82.6%)	4 (3.0%)	18 (13.6%)	1 (0.8%)	0.04
FSH/LH deficiency n, (%)	98 (74.2%)	3 (2.3%)	29 (22.0%)	2 (1.5%)	0.02
AVD n, (%)	132 (100%)	0 (0%)	0 (0%)	0 (0%)	
SES (n 95)					p-value
Pituitary deficiencies at baseline	No		Yes		
Changes from baseline	Stable	New Onset	Persistance	Recovery	
GH deficiency n, (%)	53 (55.8%)	6 (6.3%)	35 (36.8%)	1 (1.1%)	0.07
ACTH deficiency n, (%)	53 (55.8%)	7 (7.4%)	32 (33.7%)	3 (3.2%)	0.01
TSH deficiency n, (%)	52 (54.7%)	11 (11.6%)	31 (32.6%)	1 (1.1%)	0.02
FSH/LH deficiency n, (%)	39 (41.0%)	5 (5.3%)	47 (49.5%)	4 (4.2%)	0.01
AVD n, (%)	84 (88.4%)	1 (1.1%)	8 (8.4%)	2 (2.1%)	< 0.001

Primary Empty Sella (PES), Secondary Empty Sella (SES), Growth Hormone (GH), Adrenocorticotropic Hormone (ACTH), Thyroid-stimulating hormone (TSH), Follicle-stimulating Hormone (FSH), Luteinizing Hormone (LH), Arginine Vasopressin Deficiency (AVD)

The natural course of the disease displayed significant changes overtime when pituitary deficiencies were compared between PES and SES. In particular, pituitary deficiencies, both anterior (*p* = 0.037) and total deficiencies (*p* = 0.027), increased significantly in SES compared with PES, during the follow-up (Table 6). Accordingly, in the PES cohort, endocrine stability predominated since 83.3% of patients maintained unchanged pituitary function (Table 6). New hormonal deficiencies, in fact, were rare (≤ 4% per axis), and recovery of pre-existing pituitary deficiencies was observed only sporadically in PES (< 5%) (Table 6). Conversely, pituitary function in SES demonstrated greater variability over time, with a significantly higher incidence of newly developed corticotropic (7.4%; *p* = 0.01), thyrotropic (11.6%; *p* = 0.018), and gonadotropic (5.3%; *p* = 0.01) deficiencies, while changes in the GH axis did not reach statistical significance (Table 7). Recovery of prior deficits remained uncommon in both groups, underscoring the limited reversibility of established pituitary hormonal dysfunctions (Table 7). Besides, AVD appeared and partially resolved only in SES.

From a clinical standpoint, most symptoms remained stable throughout follow-up. Headache, asthenia, and visual disturbances showed minimal variation, and the occurrence of polyuria and polydipsia mirrored the pattern of AVD, being confined to SES (Table 7). In contrast, changes in sexual function were more frequent in SES, with both onset and partial recovery of decreased libido occurring more often (*p* = 0.031) (Table 6).

## Discussion

This study provides real-world longitudinal data from a large cohort of 258 patients with ES, with a median follow-up of nearly five years, allowing a comprehensive comparison between PES and SES over time. The findings clarify the natural history of ES and address clinically relevant questions regarding endocrine burden and follow-up strategies, which have so far been guided by limited and conflicting evidence.

Overall, our findings confirm that PES and SES differ in terms of clinical presentation and endocrine burden in a large sample size and, most importantly, in the longitudinal design with a relatively long follow-up. In particular, SES was associated with a higher prevalence and severity of pituitary hormone deficiencies, reflecting irreversible structural damage [3, 6, 11], whereas PES was more frequently detected incidentally and characterized by preserved endocrine function [10–12]. These results support and extend prior observations, reinforcing the distinction between the two entities from a clinical and pathophysiological perspective. In this context, our study provides real-world evidence supporting the stability of endocrine function in PES and the progressive nature of pituitary dysfunction in SES.

Consistent with previous reports, ES was diagnosed predominantly in the fifth decade of life, with a clear female predominance and a higher prevalence of PES [1, 2, 5, 13]. PES accounted for 61.6% of cases, while SES represented 38.4%, proportions comparable to those reported in other multicenter cohorts [5, 11]. Endocrine dysfunction was common, with 41.1% of patients presenting at least one pituitary hormone deficiency, a prevalence consistent with the wide range reported in the literature [2, 5, 11, 12].

The gonadotropic axis was the most frequently affected in both PES and SES, followed by other anterior pituitary axes, while AVD was rare and occurred exclusively in SES, as previously reported [11]. Similarly, the lack of association between the radiological extent of ES (partial vs. complete) and endocrine dysfunction is consistent with existing data, suggesting that imaging alone is not a reliable predictor of functional impairment [12, 14]. However, the small number of partial ES in this cohort does not allow definitive conclusion to be drawn.

This clinical profile may be related to the different underlying mechanisms—mechanical and functional in PES, destructive in SES. In PES, the pituitary gland is typically flattened by CSF pressure and diaphragm incompetence, leading to stalk stretching and transient dysregulation of hypothalamic inhibition. This mechanism may explain the mild hyperprolactinemia and isolated hormonal deficiencies seen in our and other studies [5, 10, 12]. Conversely, SES arises from irreversible glandular destruction due to apoplexy, surgery, irradiation, or trauma, explaining the higher frequency of multiple anterior and posterior pituitary deficiencies [1, 2, 4, 11]. Accordingly, the preservation of pituitary function (found in 72.3% of PES compared to 37.4% of SES) together with the AVD only seen in SES and symptoms related to the involvement of the extrasellar region (visual field defects) mostly occurring in SES underscores the distinction between these entities in terms of the degree of pituitary and neurological injury.

An important aspect that deserves consideration is the intrinsic difference in the clinical context in which PES and SES are diagnosed. SES patients are typically evaluated due to known or suspected pituitary disease, whereas PES is often identified incidentally. This difference may introduce a degree of selection bias and contribute to the observed disparities in endocrine burden. This limitation should be taken into account when interpreting the results. At the same time, this distinction reflects real-world clinical practice and represents an inherent feature of these conditions.

Sex-related differences were also evident. Women more frequently presented with PES and preserved endocrine function, whereas men showed a higher prevalence of multiple hormonal deficiencies, confirming male sex as a predictor of hypopituitarism in ES [8, 10–12, 15]. The higher rate of incidental diagnosis in women likely reflects both the female predominance of PES and gender differences in indications for neuroimaging in relation to the higher prevalence of headache and hemicrania [16, 17].

The analysis of determinants of hypopituitarism further supports the role of SES, male sex, older age, and clinical presentation [12] as key predictors of more severe endocrine involvement, while BMI [11, 12] and radiological classification [13, 14] were not significant contributors. These findings are in line with previous studies, although some discrepancies across the literature may be related to differences in study design, diagnostic approaches in terms of diagnostic criteria and the use of dynamic testing [15, 18].

Importantly, the longitudinal analysis represents a key strength of this study. While cross-sectional differences between PES and SES have been previously described, long-term comparative data remain limited. In this context, our findings provide additional evidence that endocrine function in PES is largely stable over time, whereas SES is associated with a higher risk of progression of pituitary dysfunction, confirming that pituitary dysfunction may progress in this subgroup [10–12]. Recovery of pituitary function was uncommon in both groups, underscoring the limited reversibility of established pituitary injury [19]. Although not entirely unexpected, this observation is clinically relevant, as it supports a differentiated follow-up strategy based on etiology.

Despite limitations inherent to the retrospective design and possible referral bias, several elements strengthen the robustness of our findings, including the relatively large sample size, the use of a standardized institutional database, and the consistent endocrine assessment performed at a single tertiary center.

From a clinical perspective, our data support the need for a comprehensive baseline endocrine evaluation in all patients with ES. However, follow-up strategies should be tailored according to etiology. In particular, the stability observed in PES suggests that less intensive follow-up may be appropriate in selected patients, whereas closer monitoring is warranted in SES due to the higher risk of progression. We suggest that eighteen months might be a valuable interval between follow-up visits in case of PES, especially in patients having received an incidental diagnosis. This interval should be personalized according to patient’s clinical conditions being extended in asymptomatic patients after 3–5 years of follow-up and no changes of pituitary function recorded or abbreviated in case of occurrence of new symptoms or radiological progression. The proposed follow-up interval should be interpreted as a pragmatic suggestion rather than a formal recommendation and should be individualized based on clinical context.

In conclusion, although PES and SES share similar radiological features, they differ substantially in clinical context, endocrine burden, and longitudinal course. This study provides additional long-term data that may help guide a more personalized approach to the management of patients with ES, to optimize patient management, prevent delayed diagnosis of evolving hypopituitarism in SES, and avoid unnecessary follow-up in PES.

## Supplementary Information

Below is the link to the electronic supplementary material.

Supplementary Material 1

## Data Availability

All datasets generated during and/or analyzed during the current study are not publicly available but are available from the corresponding author on reasonable request.
