# Supplementary material for: Changes overtime in primary and secondary empty sella: a comparative real-world study
Source: J Endocrinol Invest. 2026 Jun 8;49(9):2255–65. doi: 10.1007/s40618-026-02931-2 (PMC13498630; doi:10.1007/s40618-026-02931-2)
Supplement: Supplementary file 1 [file 40618_2026_2931_MOESM1_ESM.docx]

**Supplemental material**

*Hormonal assays*

Biochemical data collected at each visit were obtained from a single laboratory (Department of Laboratory Medicine and Pathology, Azienda USL of Modena, Ospedale Civile di Baggiovara, Modena, Italy). Blood samples were collected at 8:00 AM after an overnight fasting. For prolactin blood sample was drawn after 30 minutes agocannula.

Serum total testosterone (TT) was detected by immunochemiluminescence (CI) (Architect 2nd Generation Testosterone - Abbott, USA), with an intra-assay coefficient of variation < 10%.

Serum estradiol (E2) was measured by Chemiluminescent Microparticle Immunoassay (Architect platform - Abbott Laboratories), with a sensitivity of 0.6 pg/mL (2.2 pmol/L) and the lowest standard at 1.5 pg/mL, linearity to 150 pg/mL, and an ED50 of 20 pg/mL.

Serum sex hormone binding globulin (SHBG) was assessed by CI (Architect, Abbott GmbH &Co, Germany) with both inter- and intra-assay coefficients of variation of 10.0%.

Serum free testosterone (cfT) was calculated from SHBG and TT using the validated Vermeulen equation (1).

Serum LH and FSH were evaluated through chemiluminescent immunoassay (Architect, Abbott GmbH & Co, Germany) with an inter- and intra-assay coefficients of variation less than or equal to 10%.

Serum IGF-1 was assessed by chemiluminescence immuno-assay (CLIA, Diasorin), with inter-assay coefficient of variation <8.5% before July 29, 2014, and <9.6% afterwards. Serum IGFBP-3 was measured by enzyme immuno-assay (Mediagnost), with inter-assay coefficient of variation <5.7% before May 3, 2022, and by chemiluminescence immuno-assay (iSYS IDS) with inter-assay coefficient of variation ≤7.2% afterwards.

*Central adrenal insufficiency (AI)*

Adrenal axis was studied measuring serum cortisol and ACTH levels at 8–9 AM after an overnight fasting. Low or low-normal basal ACTH and cortisol levels (≤ 3 µg/dL) were diagnostic of AI; cortisol level ≥15 µg/dL likely excluded AI diagnosis (2).

A high dose corticotropin stimulation test was performed when morning cortisol values were between 3 and 15 µg/dL to diagnose AI by injecting tetracosactide (Synacthen 250 μg/1 mL, Alfasigma S.p.A. Italy) as a bolus intravenously. Blood samples for cortisol assay were collected at 0, 30 and 60 min. Peak cortisol levels <18.1 µg/dL (500 nmol/L) at 30 or 60 minutes indicated AI (2).

*Central hypothyroidism (CH)*

CH was diagnosed in the presence of an fT4 level below the laboratory reference range in conjunction with a low, normal, or mildly elevated TSH in the setting of pituitary disease (2).

*Central hypogonadism*

The diagnosis of central hypogonadism in men was established by the presence in two occasions of total testosterone levels below 3.5 ng/ml, accompanied by clinical symptoms and signs of testosterone deficiency, with inappropriately normal/low gonadotropins, according to the diagnostic criteria of clinical guidelines on male hypogonadism (3).

Serum LH and FSH levels were also measured after the administration of 100 µg of Gonadotropin-releasing hormone (GnRH) (100 g iv Gonadorelin, Ferring Pharmaceuticals Ltd., Kiel, Germany), with blood samples collected at 0, 30, 45, 60 and 90 minutes as previously described (4).

Central hypogonadism in women was diagnosed in the presence of oligomenorrhea or amenorrhea with persistent low estradiol and inappropriately low/normal FSH and LH levels. In postmenopausal women, the lack of elevated FSH and LH was sufficient to establish gonadotroph dysfunction (2).

*Growth hormone (GH)-IGF-1 axis*

GH/IGF-1 axis was investigated through serum IGF-1 and Insulin-Growth-Factor-Binding Protein 3 (IGFBP-3).

We did not perform dynamic testing in patients with clear-cut features of growth hormone deficiency (GHD) and at least three other documented pituitary hormone deficits associated with serum IGF-1 below normal range for age (12). For all the other subjects with low or reduced age adjusted IGF-1 levels a standardized Growth hormone–releasing hormone (GHRH) + Arginine (Arg) testing was performed (GHRH 1–29; GEREF, Serono, Italy; 1 μg/Kg i.v. at 0 min; arginine clorhydrate, 0.5 g/kg i.v. for 30 min, from 0 min to +30 min, up to a dose of 30 g). Blood samples were obtained every 15 min from 0 min to +60 min, then every 30 min till 120 min, as previously standardized (5, 6). GH peak response to GHRH+Arg test was evaluated according to several cut-off values available. The cut-off of 9 μg/L was used for the diagnosis of GHD in patients with a normal weight (6, 7), the cut-off of 8.0 μg/L and of 4.2 μg/L for overweight and obese patients, respectively (6). According to expert opinions on good clinical practice (8, 9) and clinical guidelines on GHD in adults (10, 11), GHRH + Arg was performed always after 6 months from starting other replacement treatments in case of multiple pituitary deficits and the correction of hypogonadism.

After the discontinuation of GHRH, our clinic has adopted the glucagon test in place of the GHRH+Arg protocol; however, no patients met the criteria for a dynamic test during this period.

In detail, in PES group 20 patients were diagnosed with GHD: 9 patients (45%) based on the presence of >3 pituitary hormone deficiencies and low IGF-1 levels, 11 patients (55%) underwent dynamic stimulation testing. In SES group 36 patients had GHD: 20 patients (55.5%) based on >3 pituitary hormone deficiencies and low IGF-1, the remaining 16 patients (44.5%) had their diagnosis through dynamic stimulation tests.

*Arginine Vasopressin Deficiency (AVD) or central diabetes insipidus*

The diagnosis of AVD was established in the presence of polyuria (more than 50 mL/kg of body weight per 24 hours, i.e., >3.5 L/day in a 70-kg individual) and polydipsia, associated with hypernatremia and increased plasma osmolality (>295 mOsm/L), in the absence of glycosuria (2). Plasma osmolality was calculated: 2 × serum sodium (mEq/L) + blood urea nitrogen (mg/dL)/2.8 + glucose (mg/dL)/18 (normal range: 285–295 mOsm/L).

In our cohort, AVD was identified exclusively in a subgroup of 10 patients with SES as a post-operative complication. The diagnosis was established based on the rapid onset of the above signs/symptoms; given the acute post-surgical context and the overt biochemical presentation, confirmatory tests such as the water deprivation test were deliberately avoided to prevent the risk of severe dehydration and acute hypernatremic complications. Copeptin measurement was not available at our center during the study period.

*Prolactin imbalance*

Hypoprolactinemia was defined as impaired lactotroph function characterized by serum prolactin below the normal range in both sexes together with a documented failure to initiate or sustain postpartum lactation in women (12).

Hyperprolactinemia was defined by the persistent elevation of serum prolactin, confirmed by a repeated testing and after exclusion of physiologic (pregnancy, lactation, stress), pharmacological (e.g., dopamine-antagonist drugs), and analytical causes (macroprolactin; hook effect) (13).

1.	Vermeulen A, Verdonck L, Kaufman JM. A critical evaluation of simple methods for the estimation of free testosterone in serum. J Clin Endocrinol Metab. 1999;84(10):3666-72.

2.	Fleseriu M, Hashim IA, Karavitaki N, Melmed S, Murad MH, Salvatori R, et al. Hormonal Replacement in Hypopituitarism in Adults: An Endocrine Society Clinical Practice Guideline. J Clin Endocrinol Metab. 2016;101(11):3888-921.

3.	Isidori AM, Aversa A, Calogero A, Ferlin A, Francavilla S, Lanfranco F, et al. Adult- and late-onset male hypogonadism: the clinical practice guidelines of the Italian Society of Andrology and Sexual Medicine (SIAMS) and the Italian Society of Endocrinology (SIE). J Endocrinol Invest. 2022;45(12):2385-403.

4.	Rochira V, Balestrieri A, Faustini-Fustini M, Borgato S, Beck-Peccoz P, Carani C. Pituitary function in a man with congenital aromatase deficiency: effect of different doses of transdermal E2 on basal and stimulated pituitary hormones. J Clin Endocrinol Metab. 2002;87(6):2857-62.

5.	Aimaretti G, Corneli G, Bellone S, Baffoni C, Camanni F, Ghigo E. Growth hormone deficiency in the transition adolescent: should treatment be continued in adult life? J Pediatr Endocrinol Metab. 2001;14 Suppl 5:1233-42; discussion 61-2.

6.	Corneli G, Di Somma C, Baldelli R, Rovere S, Gasco V, Croce CG, et al. The cut-off limits of the GH response to GH-releasing hormone-arginine test related to body mass index. Eur J Endocrinol. 2005;153(2):257-64.

7.	Ghigo E, Aimaretti G, Gianotti L, Bellone J, Arvat E, Camanni F. New approach to the diagnosis of growth hormone deficiency in adults. Eur J Endocrinol. 1996;134(3):352-6.

8.	Schneider HJ, Aimaretti G, Kreitschmann-Andermahr I, Stalla GK, Ghigo E. Hypopituitarism. Lancet. 2007;369(9571):1461-70.

9.	Prodam F, Pagano L, Corneli G, Golisano G, Belcastro S, Busti A, et al. Update on epidemiology, etiology, and diagnosis of adult growth hormone deficiency. J Endocrinol Invest. 2008;31(9 Suppl):6-11.

10.	Molitch ME, Clemmons DR, Malozowski S, Merriam GR, Vance ML. Evaluation and treatment of adult growth hormone deficiency: an Endocrine Society clinical practice guideline. J Clin Endocrinol Metab. 2011;96(6):1587-609.

11.	Ho KK. Consensus guidelines for the diagnosis and treatment of adults with GH deficiency II: a statement of the GH Research Society in association with the European Society for Pediatric Endocrinology, Lawson Wilkins Society, European Society of Endocrinology, Japan Endocrine Society, and Endocrine Society of Australia. Eur J Endocrinol. 2007;157(6):695-700.

12.	Chung TT, Korbonits M. Hypopituitarism. In: Feingold KR, Ahmed SF, Anawalt B, Blackman MR, Boyce A, Chrousos G, et al., editors. Endotext. South Dartmouth (MA): MDText.com, Inc. Copyright © 2000-2025, MDText.com, Inc.; 2000.

13.	Melmed S, Casanueva FF, Hoffman AR, Kleinberg DL, Montori VM, Schlechte JA, et al. Diagnosis and treatment of hyperprolactinemia: an Endocrine Society clinical practice guideline. J Clin Endocrinol Metab. 2011;96(2):273-88.
